# Association of maternal sleep before and during pregnancy with preterm birth and early infant sleep and temperament

**DOI:** 10.1038/s41598-020-67852-3

**Published:** 2020-07-06

**Authors:** Kazushige Nakahara, Takehiro Michikawa, Seiichi Morokuma, Masanobu Ogawa, Kiyoko Kato, Masafumi Sanefuji, Eiji Shibata, Mayumi Tsuji, Masayuki Shimono, Toshihiro Kawamoto, Shouichi Ohga, Koichi Kusuhara, The Japan Environment and Children’s Study Group, Michihiro Kamijima, Shin Yamazaki, Yukihiro Ohya, Reiko Kishi, Nobuo Yaegashi, Koichi Hashimoto, Chisato Mori, Shuichi Ito, Zentaro Yamagata, Hidekuni Inadera, Takeo Nakayama, Hiroyasu Iso, Masayuki Shima, Youichi Kurozawa, Narufumi Suganuma, Takahiko Katoh

**Affiliations:** 1 Department of Obstetrics and Gynecology, Graduate School of Medical Sciences, Kyushu University, Fukuoka, Japan; 2 Department of Environmental and Occupational Health, School of Medicine, Toho University, Tokyo, Japan; 3 Department of Health Sciences, Graduate School of Medical Sciences, Kyushu University, Fukuoka, 812-8582 Japan; 4 Research Center for Environmental and Developmental Medical Sciences, Kyushu University, Fukuoka, Japan; 5 Department of Pediatrics, Graduate School of Medical Sciences, Kyushu University, Fukuoka, Japan; 6 Japan Environment and Children’s Study, UOEH Subunit Center, University of Occupational and Environmental Health, Kitakyushu, Fukuoka Japan; 7 Department of Obstetrics and Gynecology, School of Medicine, University of Occupational and Environmental Health, Kitakyushu, Fukuoka Japan; 8 Department of Environmental Health, School of Medicine, University of Occupational and Environmental Health, Kitakyushu, Fukuoka Japan; 9 Department of Pediatrics, School of Medicine, University of Occupational and Environmental Health, Kitakyushu, Fukuoka Japan; 10 Nagoya City University, 1 Kawasumi, Mizuho-cho, Mizuho-ku, Nagoya, Aichi 467-8601 Japan; 11 National Institute for Environmental Studies, 16-2 Onogawa, Tsukuba, 305-8506 Japan; 12 National Center for Child Health and Development, 2-10-1 Okura, Setagaya-ku, Tokyo, 157-8535 Japan; 13 Hokkaido University, Kita 8, Nishi 5, Kita-ku, Sapporo, Hokkaido, 060-0808 Japan; 14 Tohoku University, 2-1 Senryo-machi, Aoba-ku, Sendai, Miyagi 980-8575 Japan; 15 Fukushima Medical University, 1 Hikariga-oka, Fukushima-shi, Fukushima, 960-1295 Japan; 16 Chiba University, 1-33 Yayoicho, Inage Ward, Chiba-shi, Chiba 263-8522 Japan; 17 Yokohama City University, 3-9 Fukuura, Kanazawa-ku, Yokohama, Kanagawa 236-0027 Japan; 18 University of Yamanashi, 1110 Shimokato, Chuo, Yamanashi 409-3898 Japan; 19 University of Toyama, 2630 Sugitani, Toyama-shi, Toyama 930-0194 Japan; 20 Kyoto University, Yoshida-honmachi, Sakyo-ku, Kyoto, 606-8501 Japan; 21 Osaka University, 1-1 Yamadaoka, Suita, Osaka 565-0871 Japan; 22 Hyogo College of Medicine, 1-1 Mukogawa, Nishinomiya, Hyogo 663-8501 Japan; 23 Tottori University, 86 Nishi-cho, Yonago, Tottori 683-8503 Japan; 24 Kochi University, Okochokohasu, Nankoku, Kochi 783-8505 Japan; 25 Kumamoto University, 1-1-1 Honjo, Chuo-ku, Kumamoto, 860-8556 Japan

**Keywords:** Epidemiology, Risk factors

## Abstract

This study aimed to investigate the association of maternal sleep before and during pregnancy with preterm birth, infant sleep and temperament at 1 month of age. We used the data of the Japan Environment and Children’s Study, a cohort study in Japan, which registered 103,099 pregnancies between 2011 and 2014. Participants were asked about their sleep before and during pregnancy, and the sleep and temperament of their newborns at 1 month of age. Preterm birth data were collected from medical records. Maternal sleep was not associated with preterm birth, but subjective sleep quality during pregnancy was associated with late preterm birth (birth at 34–36 weeks of gestation). For example, participants with extremely light subjective depth of sleep were more likely to experience preterm birth (RR = 1.19; 95% confidence interval [CI] = 1.04–1.35). Maternal sleep both before and during pregnancy seemed to be associated with infant sleep and temperament at 1 month of age. Infants, whose mothers slept for less than 6 hours before pregnancy, tended to cry intensely (RR = 1.15; 95% CI = 1.09–1.20). Maternal sleep problems before and during pregnancy were associated with preterm birth and child sleep problems and temperament.

## Introduction

In Japan, the average sleep duration is shorter than in other countries^1^, and it has become even shorter in recent years^2^. At the same time, the rate of preterm birth in Japan has increased^3^, as has the incidence of developmental disorders^4^.

Maternal short sleep duration and sleep-disordered breathing (SDB) are associated with increased preterm birth rates^5,6^. Furthermore, maternal SDB and apnea during pregnancy affects offspring’s development, manifesting as disrupted social skills and low reading test scores^7,8^.

However, no large-scale study has examined potential associations between sleep during pregnancy and offspring’s development. Additionally, the importance of maternal sleep during various periods of pregnancy remains unclear. Maternal short sleep and SDB increase inflammatory cytokine levels^9,10^, and maternal inflammation can cause preterm birth^11,12^ and developmental disorder^13,14^. Maternal obesity also increases inflammatory cytokines^15,16^, and a study reported that maternal pre-pregnancy body mass index (BMI) affects maternal inflammatory markers to a greater extent than does late pregnancy BMI^17^. Similarly, maternal sleep before pregnancy may also be more important for obstetric outcomes and offspring development than sleep during pregnancy.

In cases of neurodevelopmental disorders, including autism spectrum disorder (ASD), abnormalities of neurodevelopment, sleep, and temperament are observed in early infancy, such as a 1–4-month-old^18,19^. This study aimed to investigate the association of maternal sleep before and during pregnancy with preterm birth, infant sleep, and temperament at 1 month of age.

## Results

The baseline characteristics of participants, along with available data on sleep duration before and during pregnancy, are shown in Table 1. The most commonly reported sleep duration was 7–8 h both before and during pregnancy.

**Table 1:** Baseline characteristics of the participants in the Japan Environment and Children’s Study (2011–2014).

	No. of women^a^	Sleep duration during preconception (h)						No. of women^a^	Sleep duration during pregnancy (h)					
		< 6	6–7	7–8	8–9	9–10	> 10		< 6	6–7	7–8	8–9	9–10	> 10
		(%)	(%)	(%)	(%)	(%)	(%)		(%)	(%)	(%)	(%)	(%)	(%)
No. of women	87,106	6,028	17,260	29,350	21,521	8,830	4,117	86,358	4,305	12,948	26,769	24,491	12,166	5,679
Age at delivery (years)														
< 25	8,499	10.3	7.7	8.0	9.3	12.4	27.1	8,465	11.7	8.6	8.0	8.5	11.1	22.7
25–29	24,039	25.5	26.8	27.6	27.9	28.7	30.3	23,838	25.4	26.3	27.2	27.7	28.6	31.6
30–34	30,809	32.0	35.4	36.5	36.5	35.3	26.6	30,591	31.3	34.3	36.3	36.7	36.7	28.8
≥ 35	23,759	32.2	30.2	28.0	26.3	23.6	16.0	23,464	31.5	30.8	28.5	27.2	23.7	16.9
Smoking habits														
Never-smokers	50,646	52.8	60.3	60.4	58.4	55.8	46.4	50,269	53.7	59.5	60.1	59.1	56.7	50.3
Ex-smokers who quit before pregnancy	20,358	20.7	21.7	23.0	24.7	26.1	24.8	20,184	21.3	21.8	22.7	24.0	25.6	24.6
Smokers during early pregnancy	15,979	26.4	18.1	16.6	16.9	18.1	28.8	15,788	25.0	18.7	17.2	16.9	17.7	25.1
Alcohol consumption														
Never-drinkers	29,955	31.9	32.8	34.2	35.8	36.0	35.7	29,702	33.0	33.6	34.1	34.8	35.3	35.1
Ex-drinkers who quit before pregnancy	16,233	17.1	16.7	17.8	19.7	22.5	21.7	15,767	17.9	16.2	17.6	18.4	20.7	21.1
Drinkers during early pregnancy	40,885	51.1	50.5	48.0	44.6	41.5	42.7	40,859	49.2	50.2	48.4	46.8	44.0	43.9
Pre-pregnancy body mass index (kg/m^2^)														
< 18.5	14,045	15.8	16.1	16.0	16.2	15.8	18.0	13,969	16.9	15.9	16.0	15.8	16.7	17.6
18.5–24.9	63,883	72.4	73.8	73.6	73.5	73.3	71.0	63,334	71.4	73.7	74.0	73.9	72.4	71.3
≥ 25.0	9,140	11.8	10.2	10.4	10.3	10.9	11.0	9,000	11.8	10.5	10.0	10.3	11.0	11.1
Parity														
0	37,911	57.8	57.6	47.3	32.1	24.1	41.0	37,640	55.3	57.0	48.7	36.5	29.1	44.0
≥ 1	48,900	42.2	42.4	52.7	67.9	75.9	59.0	48,407	44.7	43.0	51.3	63.5	70.9	56.0
Current history														
Diabetes or gestational diabetes	2,665	3.5	3.2	3.0	2.9	3.1	3.2	2,638	3.9	3.2	3.1	2.9	3.0	3.1
Hypertensive disorders in pregnancy	2,759	4.5	3.7	3.2	2.7	2.4	3.0	2,618	4.0	3.6	3.1	2.8	2.6	2.6
Intrauterine infection	612	1.0	0.8	0.7	0.6	0.5	0.8	554	0.7	0.8	0.7	0.6	0.5	0.6
History of preterm birth														
No	84,156	97.0	97.1	96.8	96.3	95.8	96.7	83,518	97.0	96.9	97.0	96.6	96.4	96.6
Yes	2,934	3.1	3.0	3.2	3.7	4.2	3.3	2,799	3.1	3.1	3.0	3.4	3.7	3.4
Infertility treatment														
No	81,311	92.8	92.0	92.7	94.1	95.8	96.4	80,638	93.3	91.7	92.7	93.7	95.2	95.9
Ovulation stimulation/artificial insemination by sperm from husband	3,158	3.9	4.4	3.9	3.4	2.5	2.1	3,109	3.8	4.4	3.9	3.5	2.8	2.5
Assisted reproductive technology	2,609	3.3	3.6	3.5	2.6	1.8	1.6	2,553	2.9	3.9	3.4	2.8	2.0	1.7
Educational background, years														
< 13	30,964	40.4	32.9	33.8	35.4	40.1	54.2	31,045	42.6	34.9	33.4	34.7	37.8	48.9
≥ 13	54,926	59.6	67.1	66.2	64.6	59.9	45.8	54,986	57.4	65.1	66.6	65.3	62.2	51.1
Household income, million Japanese-yen/year														
< 6	58,726	71.3	69.5	71.1	74.6	79.3	86.1	58,763	73.9	69.6	70.3	73.1	77.6	84.9
≥ 6	21,569	28.7	30.5	28.9	25.4	20.8	14.0	21,590	26.1	30.4	29.7	26.9	22.4	15.1
Occupation in early pregnancy														
Administrative, managerial, professional, and engineering workers	20,047	22.0	26.3	25.5	22.9	17.1	9.7	19,798	20.2	24.7	25.7	24.4	19.6	12.9
Clerical workers	14,816	18.8	21.4	19.6	14.8	10.0	6.9	14,580	17.7	21.2	19.7	16.3	12.3	8.9
Sales and service workers	19,105	25.0	20.7	20.2	22.6	24.2	30.4	18,858	25.4	20.3	20.3	22.0	24.0	28.9
Homemaker	24,024	22.3	22.0	25.3	30.6	38.8	39.8	23,688	24.2	23.8	25.1	28.2	34.5	36.1
Others	8,472	11.8	9.6	9.4	9.2	9.9	13.2	8,360	12.5	10.1	9.2	9.2	9.6	13.2
Type of delivery														
Vaginal	70,776	79.8	81.1	81.2	82.0	82.3	81.8	70,603	81.9	81.4	81.6	81.9	82.9	82.4
Caesarean	16,143	20.2	18.9	18.8	18.0	17.7	18.2	15,582	18.1	18.6	18.4	18.1	17.1	17.6
Postpartum depressive symptoms at 1 month after delivery assessed by the Edinburgh Postnatal Depression Scale														
Not depressive (score < 8)	72,482	82.2	85.2	86.3	87.2	86.7	80.7	72,137	80.1	84.7	86.0	87.3	87.1	82.7
Depressive (score ≥ 9)	11,985	17.8	14.8	13.7	12.8	13.3	19.3	11,916	19.9	15.3	14.0	12.7	12.9	17.3
Small for gestational age														
No	80,106	92.3	92.2	92.5	92.6	92.8	92.8	79,496	92.7	92.5	92.6	92.7	92.6	92.6
Yes	6,495	7.7	7.8	7.5	7.4	7.2	7.3	6,339	7.3	7.5	7.4	7.3	7.4	7.4
Infant sex														
Boys	44,592	51.1	51.0	51.0	51.4	51.6	51.6	44,161	51.1	51.1	50.9	50.9	52.4	50.9
Girls	42,510	48.9	49.0	49.0	48.6	48.5	48.4	42,194	48.9	48.9	49.1	49.1	47.6	49.1

^a^Subgroup totals do not equal the overall number because of missing data.

### Maternal sleep and preterm birth

Sleep duration and bedtime before and during pregnancy were not associated with preterm birth in this study (Table 2). However, very light sleep and very bad feeling when waking up during pregnancy were associated with late preterm birth in a multivariable model (RR for very light sleep *vs.* normal = 1.19, 95% CI = 1.04–1.35; RR for very bad feeling *vs.* normal = 1.31, 95% CI = 1.02–1.67) (Table 3).

**Table 2:** Association between sleep during preconception and preterm birth in the Japan Environment and Children’s Study (2011–2014).

	No. of participants	Preterm births		Maternal age-adjusted model			Multivariable model^a^		
		No.	%	RR	95% CI		RR	95% CI	
Preterm birth									
Sleep time (h)									
< 6	6,028	288	4.8	1.08	0.95	1.22	1.04	0.92	1.17
6–7	17,260	820	4.8	1.08	0.99	1.18	1.06	0.97	1.15
7–8	29,350	1,283	4.4	Reference			Reference		
8–9	21,521	922	4.3	0.99	0.91	1.07	1.00	0.92	1.09
9–10	8,830	353	4.0	0.93	0.83	1.05	0.93	0.83	1.04
> 10	4,117	177	4.3	1.04	0.89	1.22	1.00	0.85	1.16
Bedtime									
21:00–24:00	58,425	2,582	4.4	Reference			Reference		
24:00–03:00	26,187	1,157	4.4	1.02	0.95	1.09	1.00	0.94	1.08
Other	2,494	104	4.2	0.99	0.81	1.19	0.91	0.75	1.11
Late preterm birth (34–36 weeks)									
Sleep time (h)									
< 6	5,973	233	3.9	1.10	0.95	1.26	1.07	0.93	1.22
6–7	17,104	664	3.9	1.10	1.00	1.21	1.08	0.99	1.19
7–8	29,091	1,024	3.5	Reference			Reference		
8–9	21,352	753	3.5	1.01	0.92	1.11	1.02	0.93	1.12
9–10	8,758	281	3.2	0.93	0.81	1.06	0.92	0.81	1.05
> 10	4,080	140	3.4	1.03	0.86	1.22	0.98	0.82	1.17
Bedtime									
21:00–24:00	57,925	2,082	3.6	Reference			Reference		
24:00–03:00	25,962	932	3.6	1.02	0.94	1.10	1.02	0.94	1.10
Other	2,471	81	3.3	0.96	0.77	1.19	0.89	0.71	1.11
Early preterm birth (23–33 weeks)									
Sleep time (h)									
< 6	5,795	55	1.0	1.02	0.76	1.36	0.92	0.69	1.22
6–7	16,596	156	0.9	1.02	0.83	1.24	0.96	0.78	1.17
7–8	28,326	259	0.9	Reference			Reference		
8–9	20,768	169	0.8	0.90	0.74	1.09	0.93	0.77	1.13
9–10	8,549	72	0.8	0.95	0.73	1.23	0.97	0.74	1.26
> 10	3,977	37	0.9	1.10	0.78	1.55	1.05	0.73	1.49
Bedtime									
21:00–24:00	56,343	500	0.9	Reference			Reference		
24:00–03:00	25,255	225	0.9	1.03	0.88	1.21	0.97	0.82	1.14
Other	2,413	23	1.0	1.15	0.75	1.75	1.01	0.66	1.54

CI, confidence interval; RR, relative risk.

^a^Adjusted for maternal age at delivery, smoking habits, alcohol consumption, pre-pregnancy body mass index, parity, current history of diabetes or gestational diabetes, hypertensive disorders in pregnancy and intrauterine infection, history of preterm birth, and infertility treatment.

**Table 3:** Association between sleep during pregnancy and late preterm birth in the Japan Environment and Children’s Study (2011–2014).

	No. of participants	Preterm births		Maternal age-adjusted model			Multivariable model^a^		
		No.	%	OR	95% CI		OR	95% CI	
Sleep duration (h)									
< 6	4,305	169	3.9	1.11	0.94	1.30	1.09	0.93	1.28
6–7	12,948	469	3.6	1.02	0.92	1.14	1.00	0.90	1.12
7–8	26,769	944	3.5	Reference			Reference		
8–9	24,491	882	3.6	1.03	0.94	1.12	1.04	0.95	1.14
9–10	12,166	417	3.4	0.99	0.88	1.11	0.99	0.88	1.11
> 10	5,679	196	3.5	1.03	0.89	1.20	1.01	0.87	1.18
Bedtime									
21:00–24:00	63,212	2,298	3.6	Reference			Reference		
24:00–03:00	21,173	717	3.4	0.95	0.87	1.03	0.95	0.87	1.03
Other	1,973	62	3.1	0.89	0.69	1.14	0.86	0.68	1.10
Depth of sleep									
Very light	6,135	266	4.3	1.21	1.06	1.38	1.19	1.04	1.35
Light	36,158	1,293	3.6	1.00	0.93	1.09	1.01	0.94	1.09
Normal	34,339	1,213	3.5	Reference			Reference		
Deep	8,035	258	3.2	0.92	0.80	1.05	0.94	0.82	1.07
Very deep	1,545	42	2.7	0.78	0.58	1.06	0.76	0.56	1.02
				*p* for trend < 0.01^b^			*p* for trend < 0.01^b^		
Feeling when waking up in the morning									
Very bad	1,372	65	4.7	1.38	1.08	1.75	1.31	1.02	1.67
Bad	17,875	629	3.5	1.01	0.92	1.10	1.01	0.92	1.10
Normal	53,523	1,890	3.5	Reference			Reference		
Good	11,868	428	3.6	1.02	0.92	1.13	1.05	0.94	1.16
Very good	1,526	60	3.9	1.11	0.86	1.42	1.08	0.83	1.39
				*p* for trend = 0.62^b^			*p* for trend = 0.94^b^		

CI, confidence interval; RR, relative risk.

^a^Adjusted for maternal age at delivery, smoking habits, alcohol consumption, pre-pregnancy body mass index, parity, current history of diabetes or gestational diabetes, hypertensive disorders in pregnancy and intrauterine infection, history of preterm birth, and infertility treatment.

^b^Linear trend in the association was tested by assignment of ordinal variables for the five categories.

### Maternal sleep and infant sleep

Sleep duration and bed time before pregnancy were not associated with ≥ 5 nocturnal awakenings. However, both sleep duration and bed time were likely to be related with an infant’s tendency to sleep longer during the day than at night (Table 4).

**Table 4:** Association between sleep before pregnancy and neonatal sleep in the Japan Environment and Children’s Study (2011–2014).

	No. of participants	Outcome		Maternal age-adjusted model			Multivariable model^a^		
		No.	%	RR	95% CI		RR	95% CI	
Five or more awakenings during the night									
Sleep time (h)									
< 6	5,455	355	6.5	1.02	0.91	1.14	1.07	0.96	1.20
6–7	15,797	924	5.9	0.91	0.84	0.98	0.95	0.87	1.02
7–8	27,051	1,733	6.4	Reference			Reference		
8–9	19,855	1,316	6.6	1.04	0.97	1.11	1.00	0.93	1.07
9–10	8,116	538	6.6	1.05	0.96	1.16	0.99	0.90	1.09
> 10	3,683	225	6.1	1.03	0.90	1.17	0.98	0.86	1.13
Bedtime									
21:00–24:00	53,841	3,488	6.5	Reference			Reference		
24:00–03:00	23,870	1,450	6.1	0.96	0.90	1.02	1.05	0.98	1.12
Other	2,246	153	6.8	1.13	0.96	1.32	1.17	1.00	1.37
Sleeping longer during the day than at night									
Sleep time (h)									
6	5,439	1,287	23.7	1.24	1.17	1.31	1.18	1.12	1.25
6–7	15,734	3,401	21.6	1.14	1.09	1.18	1.10	1.06	1.15
7–8	26,996	5,141	19.0	Reference			Reference		
8–9	19,811	3,535	17.8	0.93	0.90	0.97	0.98	0.95	1.02
9–10	8,088	1,349	16.7	0.87	0.82	0.92	0.94	0.89	1.00
> 10	3,673	663	18.1	0.92	0.85	0.99	0.94	0.88	1.02
Bedtime									
21:00–24:00	53,708	9,479	17,7	Reference			Reference		
24:00–03:00	23,796	5,411	22.7	1.28	1.24	1.32	1.17	1.13	1.20
Other	2,237	486	21.7	1.20	1.11	1.30	1.13	1.04	1.22

CI, confidence interval; RR, relative risk.

^a^Adjusted for maternal age at delivery, smoking habits, alcohol consumption, pre-pregnancy body mass index, gestational age at birth, parity, infertility treatment, infant sex, small for gestational age, type of delivery, and postpartum depressive symptoms.

In the M-T2, waking up ≥ 5 times during the night was associated with maternal light sleep and feeling bad at awakening during pregnancy. Sleeping longer during the day than at night was related not only to maternal short sleep duration but also to late bed time and feeling bad at awakening (Table 5).

**Table 5:** Association between sleep during pregnancy and neonatal sleep in the Japan Environment and Children’s Study (2011–2014).

	No. of participants	No. of outcome		Maternal age adjusted model			Multivariable model^a^		
			%	RR	95% CI		RR	95% CI	
Five or more awakenings during the night									
Sleep time (h)									
< 6	3,932	245	6.2	1.03	0.90	1.17	1.07	0.94	1.22
6–7	11,999	764	6.4	1.04	0.96	1.13	1.07	0.98	1.16
7–8	24,914	1,524	6.1	Reference			Reference		
8–9	22,802	1,442	6.3	1.04	0.97	1.11	1.00	0.93	1.07
9–10	11,320	801	7.1	1.17	1.08	1.27	1.10	1.01	1.20
> 10	5,183	324	6.3	1.08	0.96	1.22	1.06	0.95	1.20
Bedtime									
21:00–24:00	58,817	3,788	6.4	Reference			Reference		
24:00–03:00	19,541	1,179	6.0	0.95	0.90	1.02	1.04	0.97	1.11
Other	1,792	133	7.4	1.19	1.01	1.41	1.19	1.00	1.41
Depth of sleep									
Very light	5,567	436	7.8	1.29	1.17	1.43	1.27	1.14	1.40
Light	33,580	2,224	6.6	1.09	1.03	1.15	1.07	1.01	1.14
Normal	31,909	1,929	6.1	Reference			Reference		
Deep	7,510	418	5.6	0.92	0.83	1.02	0.94	0.85	1.04
Very deep	1,450	84	5.8	0.98	0.79	1.21	1.00	0.81	1.24
				*p* for trend < 0.01^b^			*p* for trend < 0.01^b^		
Feeling when waking up in the morning									
Very bad	1,240	112	9.0	1.52	1.27	1.81	1.53	1.28	1.84
Bad	16,514	1,131	6.9	1.12	1.05	1.19	1.12	1.05	1.20
Normal	49,715	3,073	6.2	Reference			Reference		
Good	11,085	689	6.2	1.00	0.92	1.08	1.01	0.93	1.09
Very good	1,422	81	5.7	0.92	0.74	1.14	0.95	0.76	1.17
				*p* for trend < 0.01^b^			*p* for trend < 0.01^b^		
Sleeping longer during the day than at night									
Sleep time (h)									
< 6	3,914	915	23.4	1.19	1.12	1.27	1.16	1.09	1.24
6–7	11,958	2,663	22.3	1.14	1.10	1.19	1.11	1.06	1.16
7–8	24,868	4,843	19.5	Reference			Reference		
8–9	22,738	4,147	18.2	0.94	0.90	0.97	0.97	0.94	1.01
9–10	11,288	1,898	16.8	0.86	0.82	0.90	0.92	0.88	0.97
> 10	5,167	964	18.7	0.93	0.88	0.99	0.96	0.90	1.03
Bedtime									
21:00–24:00	58,673	10,533	18.0	Reference			Reference		
24:00–03:00	19,499	4,516	23.2	1.28	1.24	1.32	1.18	1.14	1.22
Other	1,783	381	21.4	1.17	1.07	1.29	1.15	1.05	1.26
Depth of sleep									
Very light	5,550	1,162	20.9	1.09	1.03	1.15	1.08	1.02	1.15
Light	33,500	6,397	19.1	0.99	0.96	1.02	1.00	0.97	1.03
Normal	31,812	6,139	19.3	Reference			Reference		
Deep	7,489	1,427	19.1	0.99	0.94	1.04	0.96	0.92	1.02
Very deep	1,448	286	19.8	1.02	0.91	1.13	0.99	0.89	1.10
				*p* for trend = 0.15^b^			*p* for trend = 0.02^b^		
Feeling when waking up in the morning									
Very bad	1,237	300	24.1	1.24	1.12	1.37	1.21	1.09	1.34
Bad	16,468	3,329	20.1	1.05	1.02	1.09	1.04	1.00	1.07
Normal	49,582	9,472	19.0	Reference			Reference		
Good	11,056	2,094	18.9	1.00	0.95	1.04	0.99	0.95	1.03
Very good	1,416	258	18.1	0.95	0.84	1.06	0.93	0.83	1.05
				*p* for trend < 0.01^b^			*p* for trend < 0.01^b^		

CI, confidence interval; RR, relative risk.

^a^Adjusted for maternal age at delivery, smoking habits, alcohol consumption, pre-pregnancy body mass index, gestational age at birth, parity, infertility treatment, infant sex, small for gestational age, type of delivery, and postpartum depressive symptoms.

^b^Linear trend in the association was tested by assignment of ordinal variables for the five categories.

In the subanalysis of participants who slept 7–9 h before pregnancy, maternal short sleep and bed time after midnight were associated with longer day sleeps. Similarly, in the group of participants who slept 7–9 h during pregnancy, maternal short sleep before pregnancy was associated with more awakenings during the night and longer day-time sleep durations (Table S1).

### Maternal sleep and infant temperament

In the M-T1, sleeping < 7 h and going to bed after midnight were associated with infants with bad mood, frequent crying for long periods, and intense crying (Table 6).

**Table 6:** Association between sleep during preconception and neonatal irritability in the Japan Environment and Children’s Study (2011–2014).

	No. of participants	Outcome		Maternal age-adjusted model			Multivariable model^a^		
		No.	%	RR	95% CI		RR	95% CI	
Bad mood									
Sleep time (h)									
< 6	5,603	469	8.4	1.33	1.21	1.47	1.12	1.01	1.23
6–7	16,142	1,278	7.9	1.26	1.17	1.35	1.09	1.01	1.16
7–8	27,584	1,735	6.3	Reference			Reference		
8–9	20,238	966	4.8	0.76	0.70	0.82	0.97	0.90	1.05
9–10	8,308	356	4.3	0.68	0.61	0.76	1.05	0.94	1.17
> 10	3,804	239	6.3	1.00	0.87	1.14	1.17	1.02	1.33
Bedtime									
21:00–24:00	54,935	2,803	5.1	Reference			Reference		
24:00–03:00	24,434	2,084	8.5	1.68	1.59	1.77	1.12	1.06	1.19
Other	2,310	156	6.8	1.35	1.15	1.57	1.10	0.94	1.29
Frequent crying, for long periods									
Sleep time (h)									
< 6	5,587	1,255	22.5	1.28	1.21	1.35	1.17	1.11	1.24
6–7	16,093	3,332	20.7	1.18	1.14	1.23	1.09	1.05	1.13
7–8	27,534	4,810	17.5	Reference			Reference		
8–9	20,205	3,013	14.9	0.85	0.82	0.89	0.98	0.94	1.02
9–10	8,299	1,066	12.8	0.74	0.69	0.78	0.91	0.86	0.97
> 10	3,800	619	16.3	0.94	0.87	1.01	1.01	0.94	1.09
Bedtime									
21:00–24:00	54,840	8,530	15.6	Reference			Reference		
24:00–03:00	24,365	5,178	21.3	1.38	1.33	1.42	1.09	1.06	1.13
Other	2,313	387	16.7	1.09	1.00	1.20	0.96	0.88	1.06
Intense crying									
Sleep time (h)									
< 6	5,592	1,448	25.9	1.30	1.23	1.36	1.15	1.09	1.20
6–7	16,114	3,886	24.1	1.21	1.17	1.25	1.08	1.04	1.12
7–8	27,546	5,490	19.9	Reference			Reference		
8–9	20,201	3,234	16.0	0.80	0.77	0.84	0.97	0.93	1.01
9–10	8,295	1,149	13.9	0.69	0.65	0.74	0.95	0.89	1.00
> 10	3,800	671	17.7	0.88	0.81	0.94	0.98	0.91	1.06
Bedtime									
21:00–24:00	54,833	9,350	17.1	Reference			Reference		
24:00–03:00	24,402	6,082	24.9	1.46	1.42	1.51	1.07	1.04	1.10
Other	2,313	446	19.3	1.14	1.04	1.24	0.97	0.89	1.05

CI, confidence interval; RR, relative risk.

^a^Adjusted for maternal age at delivery, smoking habits, alcohol consumption, pre-pregnancy body mass index, gestational age at birth, parity, infertility treatment, infant sex, small for gestational age, type of delivery, and postpartum depressive symptoms.

In the M-T2, we observed a relationship between bad mood and maternal short sleep, late bed time, light sleep, and feeling bad on awakening. Frequent crying for long periods was likely to be associated with short sleep, late bed time, light sleep, and feeling bad on awakening. Intense crying was also related to short sleep, late bed time, light sleep, and feeling bad on awakening (Table 7).

**Table 7:** Association between sleep during pregnancy and neonatal temperament in the Japan Environment and Children’s Study (2011–2014).

	No. of participants	Outcome		Maternal age-adjusted model			Multivariable model^a^		
		No.	%	RR	95% CI		RR	95% CI	
Bad mood									
Sleep duration (h)									
< 6	4,049	373	9.2	1.40	1.26	1.56	1.22	1.09	1.36
6–7	12,252	959	7.8	1.19	1.10	1.29	1.06	0.99	1.15
7–8	25,432	1,671	6.6	Reference			Reference		
8–9	23,266	1,215	5.2	0.79	0.74	0.85	0.97	0.91	1.04
9–10	11,551	560	4.9	0.74	0.67	0.81	1.03	0.94	1.13
> 10	5,343	301	5.6	0.85	0.76	0.96	0.95	0.85	1.07
Bedtime									
21:00–24:00	60,030	3,214	5.4	Reference			Reference		
24:00–03:00	20,013	1,759	8.8	1.64	1.55	1.74	1.15	1.09	1.22
Other	1,850	106	5.7	1.08	0.89	1.30	0.96	0.79	1.16
Depth of sleep									
Very light	5,740	480	8.4	1.47	1.33	1.61	1.46	1.32	1.61
Light	34,277	2,172	6.3	1.11	1.05	1.18	1.14	1.07	1.21
Normal	32,606	1,861	5.7	Reference			Reference		
Deep	7,656	447	5.8	1.02	0.92	1.13	0.95	0.86	1.05
Very deep	1,475	107	7.3	1.27	1.05	1.53	1.20	1.00	1.45
				*p* for trend < 0.01^b^		*p* for trend < 0.01^b^			
Feeling when waking up in the morning									
Very bad	1,269	131	10.3	1.82	1.54	2.15	1.57	1.33	1.86
Bad	16,920	1,353	8.0	1.41	1.32	1.50	1.29	1.21	1.37
Normal	50,810	2,886	5.7	Reference			Reference		
Good	11,265	612	5.4	0.96	0.88	1.04	0.92	0.85	1.01
Very good	1,448	83	5.7	1.01	0.82	1.25	0.99	0.80	1.22
				*p* for trend < 0.01^b^		*p* for trend < 0.01^b^			
Frequent crying, for long periods									
Sleep duration (h)									
< 6	4,036	898	22.3	1.24	1.16	1.32	1.15	1.08	1.22
6–7	12,229	2,587	21.2	1.18	1.13	1.23	1.09	1.05	1.14
7–8	25,373	4,543	17.9	Reference			Reference		
8–9	23,209	3,609	15.6	0.87	0.84	0.90	0.96	0.93	1.00
9–10	11,551	1,669	14.5	0.81	0.77	0.85	0.97	0.92	1.02
> 10	5,333	862	16.2	0.91	0.85	0.97	0.97	0.91	1.04
Bedtime									
21:00–24:00	59,915	9,559	16.0	Reference			Reference		
24:00–03:00	19,970	4,253	21.3	1.34	1.30	1.39	1.09	1.05	1.13
Other	1,846	356	19.3	1.22	1.11	1.34	1.15	1.04	1.26
Depth of sleep									
Very light	5,727	1,194	20.9	1.27	1.20	1.35	1.24	1.18	1.31
Light	34,222	6,148	18.0	1.10	1.07	1.14	1.11	1.07	1.14
Normal	32,529	5,294	16.3	Reference			Reference		
Deep	7,642	1,270	16.6	1.02	0.97	1.08	0.98	0.93	1.04
Very deep	1,474	238	16.2	1.00	0.88	1.12	0.95	0.84	1.07
				*p* for trend < 0.01^b^		*p* for trend < 0.01^b^			
Feeling when waking up in the morning									
Very bad	1,262	301	23.9	1.44	1.30	1.59	1.27	1.15	1.41
Bad	16,880	3,515	20.8	1.25	1.21	1.30	1.17	1.13	1.21
Normal	50,721	8,456	16.7	Reference			Reference		
Good	11,243	1,668	14.8	0.89	0.85	0.93	0.88	0.84	0.92
Very good	1,446	191	13.2	0.79	0.69	0.90	0.78	0.68	0.89
				*p* for trend < 0.01^b^		*p* for trend < 0.01^b^			
Intense crying									
Sleep duration (h)									
< 6	4,043	1,053	26.1	1.25	1.18	1.33	1.15	1.08	1.21
6–7	12,226	2,958	24.2	1.17	1.12	1.22	1.07	1.03	1.11
7–8	25,404	5,256	20.7	Reference			Reference		
8–9	23,224	3,941	17.0	0.82	0.79	0.85	0.95	0.92	0.99
9–10	11,542	1,807	15.7	0.76	0.72	0.79	0.98	0.93	1.02
> 10	5,327	946	17.8	0.85	0.80	0.91	0.94	0.88	1.00
Bedtime									
21:00–24:00	59,939	10,555	17.6						
24:00–03:00	19,982	5,025	25.2	1.43	1.39	1.47	1.08	1.05	1.12
Other	1,845	381	20.7	1.17	1.07	1.29	1.09	1.00	1.19
Depth of sleep									
Very light	5,746	1,206	21.0	1.11	1.06	1.18	1.11	1.05	1.17
Light	34,306	6,813	19.9	1.06	1.03	1.09	1.08	1.05	1.12
Normal	32,669	6,129	18.8	Reference			Reference		
Deep	7,664	1,545	20.2	1.07	1.02	1.13	1.02	0.97	1.07
Very deep	1,481	294	19.9	1.05	0.95	1.17	1.00	0.91	1.11
				*p* for trend < 0.01^b^		*p* for trend < 0.01^b^			
Feeling when waking up in the morning									
Very bad	1,266	302	23.9	1.28	1.16	1.42	1.16	1.05	1.28
Bad	16,887	3,853	22.8	1.23	1.19	1.27	1.16	1.12	1.19
Normal	50,737	9,417	18.6	Reference			Reference		
Good	11,251	2,093	18.6	1.00	0.96	1.05	0.98	0.94	1.02
Very good	1,445	255	17.7	0.95	0.85	1.06	0.93	0.83	1.04
				*p* for trend < 0.01^b^		*p* for trend < 0.01^b^			

CI, confidence interval; RR, relative risk.

^a^Adjusted for maternal age at delivery, smoking habits, alcohol consumption, pre-pregnancy body mass index, gestational age at birth, parity, infertility treatment, infant sex, small for gestational age, type of delivery, and postpartum depressive symptoms.

^b^Linear trend in the association was tested by assignment of ordinal variables for the five categories.

In the subanalysis of participants who slept 7–9 h before pregnancy, maternal short sleep during pregnancy was associated with bad mood in infants. Similarly, in the group of participants who slept 7–9 h during pregnancy, maternal late bed time before pregnancy was associated with bad mood in infants (Table S2).

## Discussion

This study investigated whether maternal sleep before and during pregnancy was associated with preterm birth, infants sleep and temperament at 1 month of age, using data from a nationwide large-scale cohort study.

In this study, preterm birth was only associated with the quality of maternal sleep during pregnancy. Previous studies reported an association between preterm birth and subjective maternal sleep quality^9,20^. Subjective sleep depth and mood on awakening may reflect maternal SDB or depression^21,22^, both of which are known to increase preterm birth rates^6,20^. Interventions to improve sleep depth and mood on awakening could reduce preterm birth rates.

Contrary to a previous meta-analysis in 2018^5^, we found no association of sleep duration with preterm birth. One conceivable reason for this difference is the lower rate of preterm birth in Japan compared to other countries^23,24^.

This study also revealed an association of maternal sleep before and during pregnancy with infant sleep and temperament at 1 month of age. To our knowledge, no studies have previously reported similar findings. However, infant sleep disorders and temperament have been reported to increase maternal stress^25^ and relate to maternal anxiety disorder and depression^26^. As a result, it may lead to parenting abandonment and child abuse^27^. If maternal sleep before and during pregnancy could be addressed to improve infant sleep and temperament, maternal postpartum mental disease and child abuse could also be reduced.

Maternal sleep before pregnancy also seemed to be associated with some features of infant temperament and frequent night awakenings in the group of participants with adequate sleep duration (7–9 h) during pregnancy. The Centers for Disease Control and Prevention and the World Health Organization recently issued statements on the importance of preconception care^28^, without emphasising maternal sleep. We should probably pay more attention to maternal sleep not only during pregnancy but also before pregnancy.

This study had four major limitations. First, maternal obesity and depression may be confounding factors: both are associated with sleeping problems and can cause maternal inflammation^29,30^. Some epidemiological studies have also reported that maternal obesity and depression are associated with preterm birth and developmental disorders^20,31^. In this study, we showed an association between maternal sleep and preterm birth, infant sleeping problems, and temperament after adjustment for pre-pregnancy BMI and postpartum depressive symptoms. Maternal sleep may affect these outcomes. However, adjustment for maternal depression could be insufficient because depressive symptoms were investigated postpartum.

Second, information about maternal and infant sleeping problems and infant temperament were collected using a questionnaire.

Third, because of the observational study design, we were not able to demonstrate how maternal sleep affected outcomes. Evidence suggests that inflammatory response plays a role, due to maternal sleep disruption during pregnancy causing preterm birth and aberrant development of offspring; short maternal sleep duration and SDB increases inflammatory cytokine levels^9,10^. It is believed that maternal inflammation can cause preterm birth^11,12^ and developmental disorders, such as ASD^13,14^. However, this study did not analyse inflammatory markers.

Fourth, the large sample size might show a statistically significant difference even when the difference is small. Since there have been no similar reports to this work, further investigations of relationships between maternal sleep and offspring’s outcome are necessary.

In conclusion, maternal sleep problems before and during pregnancy were associated with preterm birth and infant sleep problems and temperament.

## Methods

### Research ethics

The study protocol was approved by the Ministry of Environment’s Institutional Review Board on Epidemiological Studies and by the Ethics Committee of all participating institutions: the National Institute for Environmental Studies that leads the JECS, the National Center for Child Health and Development, Hokkaido University, Sapporo Medical University, Asahikawa Medical College, Japanese Red Cross Hokkaido College of Nursing, Tohoku University, Fukushima Medical University, Chiba University, Yokohama City University, University of Yamanashi, Shinshu University, University of Toyama, Nagoya City University, Kyoto University, Doshisha University, Osaka University, Osaka Medical Center and Research Institute for Maternal and Child Health, Hyogo College of Medicine, Tottori University, Kochi University, University of Occupational and Environmental Health, Kyushu University, Kumamoto University, University of Miyazaki, and University of Ryukyu.. Written informed consent, which also included the follow-up study of children after birth, was obtained from all participants. All methods were performed in accordance with approved guidelines.

### Study participants

Data used in this study were obtained from the Japan Environment and Children’s Study (JECS), an ongoing large-scale cohort study. The JECS elucidated environmental factors that affected children’s health and development and was designed to follow-up pregnant women until their newborns grow up to 13 years old. The participants were recruited between 2011 and 2014 in 15 regions throughout Japan, and the follow-up is carried out mainly by a self-administered questionnaire. The detailed protocol has been reported elsewhere^32^. The baseline profile of participants in the JECS was reported previously^33^. Participants answered a questionnaire about lifestyle and behaviour twice during pregnancy: at recruitment (early pregnancy, M-T1) and later during mid- and late-pregnancy (M-T2). Participants also answered a questionnaire about their newborns at 1 month after delivery (M-1m).

**Figure 1:**
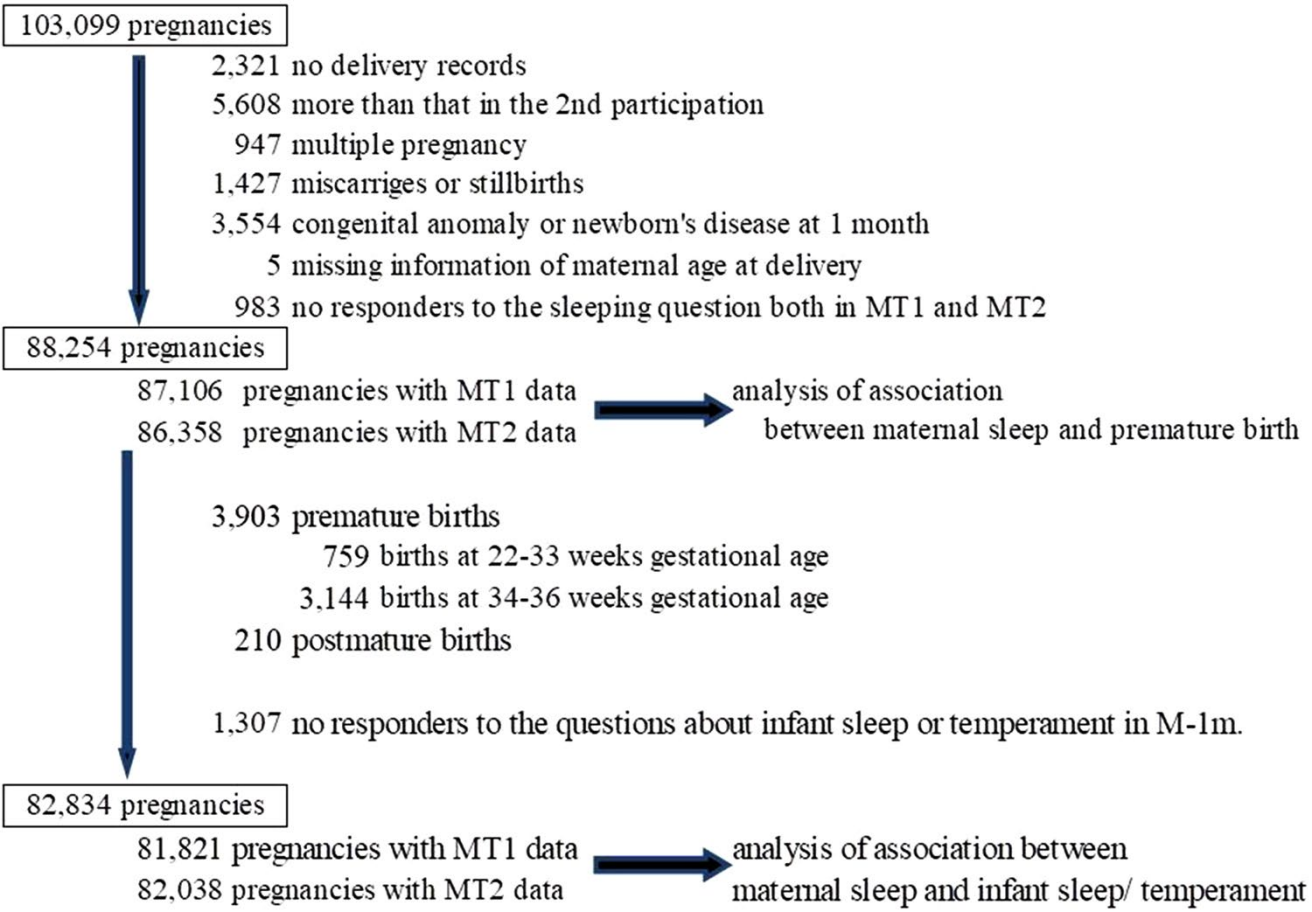
Flow chart representing the study population. M-T1: questionnaire administered at recruitment; M-T2: questionnaire administered during mid- and late-pregnancy; M-1m: questionnaire administered at 1 month after delivery.

Of all 103,099 pregnancies, we excluded 14,845 pregnancies due to the following reasons: no delivery records (n = 2,321), prior participation in the study (n = 5,608), multiple pregnancy (n = 947), miscarriage or stillbirth (n = 1,427), congenital anomaly identified by 1 month old (n = 3,554), missing information on maternal age at delivery (n = 5), and no response to the questions related to sleep at both M-T1 and M-T2 (n = 983). The remaining 88,254 participants (87,106 with M-T1 data and 86,358 with M-T2 data) were included in the analysis of the association between maternal sleep and preterm birth. Then, we further excluded preterm or post-term deliveries (n = 4,113) and participants who did not respond to the questions related to the children’s sleep and temperament at M-1m (n = 1,307). (Fig. 1) Finally, 81,821 participants with M-T1 data and 82,038 participants with M-T2 data were included in the analysis of the association between maternal sleep and infant sleep and temperament.

### Maternal sleep

In the M-T1 questionnaire, participants were asked about their sleep duration and bed time before pregnancy. We divided participants into six groups by sleep time: < 6 h, 6–7 h, 7–8 h, 8–9 h, 9–10 h, and > 10 h. Participants were also divided by bedtime: 9:00 p.m. to midnight, midnight to 3 a.m., and others.

In the M-T2 questionnaire, participants were asked about their sleep duration and bed time during pregnancy. The participants were divided into groups as described above for the M-T1. In addition, the M-T2 questionnaire included two more questions about sleep quality. One was, “How would you rate your average depth of sleep during the past month?” The other one was, “How would you rate your overall feeling when waking up in the morning during past month?” The answers to both questions were scored 1, 2, 3, 4, and 5, representing very light (bad), relatively light (bad), normal, relatively deep (good), and very deep (good), respectively.

### Preterm birth

Information on gestational age at delivery was transferred from medical records. Preterm birth was defined as birth before 37 completed weeks of gestation. In the present study, 3,903 (4.4%) pregnant women had preterm births. We divided these women into two groups: the early preterm birth group (birth before 34 completed weeks of gestation) and the late preterm birth group (birth after 34 gestational weeks). There were 759 (0.9%) women in the early preterm birth group and 3,144 (3.6%) women in the late preterm birth group. In the analysis of association between maternal sleep during pregnancy and preterm birth, we selected only late preterm birth as an outcome because some participants in the early preterm birth group did not answer M-T2 questionnaire between 22 and 28 weeks gestational age. So, the available number of participants in the early preterm group was too small to analyse.

### Outcome (infant sleep and temperament)

At 1 month after delivery, we assessed infant sleep and temperature using a parents-reported questionnaire (M-1m questionnaire). For infant sleep, participants were asked about sleep and wake times during a 24-h period for their newborns. In this analysis, we focused on two points. First, we analysed the number of nocturnal awakenings. We defined ≥ 5 awakenings as too many because a previous study reported that the average number of awakenings during the night (20:00 – 7:59) is 2.95 (range: 1.0–5.0) for 2-week-old infants^34^. Second, we analysed whether the infants slept longer during the day (08:00–19:59) or at night (20:00–7:59). We regarded longer sleeping times during the day than at night as unusual.

Also, the M-1m questionnaire included three questions about infant temperament. The first question was, “When you hold your baby, how often do you feel difficulty to hold your baby due to his/her fretting, crying, or throwing his/her head back?”; the answer options were “often,” “sometimes”, “seldom,” and “none.” Those who answered “often” were categorised as “bad mood.” The second question was, “How often and for how long does your baby cry?”; the answer options were “quite often and for long periods,” “sometimes and for short periods,” and “seldom and almost never.” Those who answered as “quite often and long” were categorised as “frequent crying, for long periods.” The third question was “Does your baby cry very hard sometimes no matter what you do to stop him/her?”; the answer options were “yes” and “no,” and those who answered “yes” were categorised as “intense crying”. These categories are the same as those in our previous study^35^.

### Covariates

Information about maternal age at delivery, smoking habits, alcohol consumption, pre-pregnancy body mass index (BMI), parity, current history of diabetes, gestational diabetes, hypertensive disorder in pregnancy and intrauterine infection, history of preterm birth, gestational age at birth, infertility treatment, infant sex, type of delivery, small for gestational age and postpartum depressive symptoms were collected via self-administered questionnaires and/or medical records. Postpartum depressive symptoms were assessed using the Edinburgh Postnatal Depression Scale (EPDS), including in the questionnaire at 1 month after delivery^36^. According to previous studies^37^, participants with a score of 9 or more were categorized as having depressive symptoms.

### Statistical analyses

We used the Poisson regression model with a robust error variance^38^ to explore the association of maternal sleep before and during pregnancy with each outcome and to estimate the relative risks (RRs) of each outcome and 95% confidence intervals (CIs). We initially adjusted for maternal age at delivery, and then further adjusted as follows. In the analysis of the association between maternal sleep and preterm birth, we adjusted for smoking habits (never-smokers, ex-smokers who quit before pregnancy, smokers during early pregnancy), alcohol consumption (never-drinkers, ex-drinkers who quit before pregnancy, drinkers during early pregnancy), pre-pregnancy BMI (< 18·5, 18·5–24·9, ≥ 25·0 kg m^2^), current history of diabetes or gestational diabetes (yes, no), hypertensive disorders in pregnancy (yes, no) and intrauterine infection (yes, no), parity (0, ≥ 1), history of preterm birth (yes, no) and infertility treatment (yes, no).

In the analysis of the association between maternal sleep and infant sleep or temperament, we adjusted for smoking habits, alcohol consumption, pre-pregnancy BMI, gestational age at birth (37, 38, 39, 40, and 41 weeks of gestation), infant sex (boys, girls), parity, infertility treatment, type of delivery (vaginal, caesarean section), small for gestational age infants (yes, no), and postpartum depressive symptoms (yes, no).

We performed a subanalysis of infant sleep or temperament in the participant groups that reported adequate sleep durations of 7–9 h both at M-T1 and M-T2 to evaluate how maternal sleep before and during pregnancy impacts the outcome of these parameters.

The dataset used for statistical analyses was the jecs-ag-20160424 dataset, which was released in June 2016, and revised in October 2016, along with the supplementary dataset jecs-ag-20160424-sp1. Stata version 14 (StataCorp LP, College Station, Texas, USA) was used for all analyses.

## Supplementary information

Supplementary file1 (DOCX 64 kb)
