# Supplementary material for: Association of maternal sleep before and during pregnancy with preterm birth and early infant sleep and temperament
Source: Sci Rep. 2020 Jul 6;10:11084. doi: 10.1038/s41598-020-67852-3 (PMC7338358; doi:10.1038/s41598-020-67852-3)
Supplement: Supplementary file 1 — Supplementary file1 (DOCX 64 kb) [file 41598_2020_67852_MOESM1_ESM.docx]

**Association of maternal sleep before and during pregnancy with preterm birth and early infant sleep and temperament**

**Supplemental materials**

Table S1

Table S2

Kazushige Nakahara^1**^, Takehiro Michikawa^2**^, Seiichi Morokuma*^3, 4^, Masanobu Ogawa^4^, Kiyoko Kato^1, 4^, Masafumi Sanefuji^4, 5^,

Eiji Shibata^6, 7^, Mayumi Tsuji^6, 8^, Masayuki Shimono^6, 9^, Toshihiro Kawamoto^6^, Shouichi Ohga^5^, Koichi Kusuhara^6, 9^ and the Japan Environment and Children’s Study Group

^**^These authors contributed equally to this work.

| **Table S1.** Association between maternal sleep duration before or during pregnancy and infant sleep: Sub-analysis of the Japan Environment and Children’s Study (2011–2014) | | | | | | | | | | | | | | | |
| --- | --- | --- | --- | --- | --- | --- | --- | --- | --- | --- | --- | --- | --- | --- | --- |
|  |  |  | **Sleep during pregnancy in participants  sleeping for 7 to 9 hours before pregnancy** | | | | | | **Sleep before pregnancy in participants  sleeping for 7 to 9 hours during pregnancy** | | | | | | |
|  |  |  | **No. of participants** | **Outcome** | | **Multivariable model*** | | |  | **No. of participants** | **Outcome** | | **Multivariable model*** | | |
|  |  |  |  | **No.** | **%** | **RR** | **95% CI** | |  |  | **No.** | **%** | **RR** | **95% CI** | |
| **Five or more awakenings during the night** | | | | |  |  |  |  |  |  |  |  |  |  |  |
|  | **Sleep duration, hours** | | |  |  |  |  |  |  |  |  |  |  |  |  |
|  |  | **<6** | 668 | 50 | 7.5 | **1.28** | 0.97 | 1.68 |  | 1,543 | 111 | 7.2 | **1.21** | 1.00 | 1.46 |
|  |  | **6–7** | 3,780 | 265 | 7.0 | **1.13** | 0.99 | 1.28 |  | 7,426 | 408 | 5.5 | **0.89** | 0.80 | 1.00 |
|  |  | **7–8** | 17,063 | 1,083 | 6.4 | **Reference** | |  |  | 20,452 | 1,298 | 6.4 | **Reference** | |  |
|  |  | **8–9** | 17,144 | 1,093 | 6.4 | **0.98** | 0.90 | 1.06 |  | 13,755 | 878 | 6.4 | **0.98** | 0.90 | 1.07 |
|  |  | **9–10** | 5,835 | 407 | 7.0 | **1.07** | 0.96 | 1.19 |  | 3,001 | 186 | 6.2 | **0.97** | 0.83 | 1.13 |
|  |  | **>10** | 2,025 | 129 | 6.4 | **1.08** | 0.91 | 1.30 |  | 1,067 | 60 | 5.6 | **0.97** | 0.75 | 1.25 |
|  | **Bedtime** | |  |  |  |  |  |  |  |  |  |  |  |  |  |
|  |  | **21:00–24:00** | 37,874 | 2,475 | 6.5 | **Reference** | |  |  | 34,865 | 2,189 | 6.3 | **Reference** | |  |
|  |  | **24:00–03:00** | 7,965 | 499 | 6.3 | **1.06** | 0.97 | 1.17 |  | 11,575 | 698 | 6.0 | **1.06** | 0.97 | 1.16 |
|  |  | **Other** | 676 | 53 | 7.8 | **1.26** | 0.97 | 1.64 |  | 864 | 56 | 6.5 | **1.18** | 0.90 | 1.53 |
| **Sleeping longer during the day than at night** | | | | | |  |  |  |  |  |  |  |  |  |  |
|  | **Sleep duration, hours** | | |  |  |  |  |  |  |  |  |  |  |  |  |
|  |  | **<6** | 667 | 164 | 24.6 | **1.25** | 1.09 | 1.44 |  | 1,540 | 359 | 23.3 | **1.19** | 1.08 | 1.31 |
|  |  | **6–7** | 3,772 | 793 | 21.0 | **1.09** | 1.02 | 1.17 |  | 7,404 | 1,568 | 21.2 | **1.10** | 1.05 | 1.16 |
|  |  | **7–8** | 17,038 | 3,171 | 18.6 | **Reference** | |  |  | 20,415 | 3,771 | 18.5 | **Reference** | |  |
|  |  | **8–9** | 17,101 | 3,068 | 17.9 | **1.00** | 0.95 | 1.04 |  | 13,724 | 2,468 | 18.0 | **1.02** | 0.97 | 1.07 |
|  |  | **9–10** | 5,819 | 1039 | 17.9 | **1.00** | 0.94 | 1.06 |  | 2,990 | 536 | 17.9 | **1.02** | 0.94 | 1.11 |
|  |  | **>10** | 2,020 | 374 | 18.5 | **0.97** | 0.88 | 1.07 |  | 1,064 | 202 | 19.0 | **0.98** | 0.87 | 1.12 |
|  | **Bedtime** | |  |  |  |  |  |  |  |  |  |  |  |  |  |
|  |  | **21:00–24:00** | 37,843 | 6,712 | 17.7 | **Reference** | |  |  | 34,787 | 6,143 | 17.7 | **Reference** | |  |
|  |  | **24:00–03:00** | 7,953 | 1,759 | 22.1 | **1.14** | 1.09 | 1.20 |  | 11,549 | 2,573 | 22.3 | **1.15** | 1.10 | 1.20 |
|  |  | **Other** | 673 | 147 | 21.8 | **1.19** | 1.03 | 1.38 |  | 860 | 199 | 23.1 | **1.18** | 1.04 | 1.34 |
| CI, confidence interval; RR, relative risk | | | | | | | | | | | | | | | |
| *Adjusted for maternal age at delivery, smoking habits, alcohol consumption, pre-pregnancy body mass index, gestational age at birth, parity, infertility treatment, infant sex, small for gestational age, type of delivery, postpartum depressive symptoms, educational background, household income, and occupation. | | | | | | | | | | | | | | | |

| **Table S2.** Association between maternal sleep duration before or during pregnancy and infant sleep and temperament: Sub-analysis  of the Japan Environment and Children’s Study (2011–2014) | | | | | | | | | | | | | | | |
| --- | --- | --- | --- | --- | --- | --- | --- | --- | --- | --- | --- | --- | --- | --- | --- |
|  |  |  | **Sleep during pregnancy in participants  sleeping for 7 to 9 hours before pregnancy** | | | | | |  | **Sleep before pregnancy in participants  sleeping for 7 to 9 hours during pregnancy** | | | | | |
|  |  |  | **No. of participants** | **Outcome** | | **Multivariable model*** | | |  | **No. of participants** | **Outcome** | | **Multivariable model*** | | |
|  |  |  |  | **No.** | **%** | **RR** | **95% CI** | |  |  | **No.** | **%** | **RR** | **95% CI** | |
| **Bad mood** | | |  |  |  |  |  |  |  |  |  |  |  |  |  |
|  | **Sleep duration, hours** | | |  |  |  |  |  |  |  |  |  |  |  |  |
|  |  | **<6** | 695 | 68 | 9.8 | **1.37** | 1.08 | 1.74 |  | 1,587 | 125 | 7.9 | **1.10** | 0.92 | 1.32 |
|  |  | **6–7** | 3,858 | 279 | 7.2 | **1.13** | 1.00 | 1.29 |  | 7,589 | 588 | 7.8 | **1.13** | 1.03 | 1.24 |
|  |  | **7–8** | 17,369 | 1,030 | 5.9 | **Reference** | |  |  | 20,834 | 1,253 | 6.0 | **Reference** | |  |
|  |  | **8–9** | 17,458 | 871 | 5.0 | **1.01** | 0.93 | 1.10 |  | 13,993 | 648 | 4.6 | **0.99** | 0.91 | 1.09 |
|  |  | **9–10** | 5,942 | 292 | 4.9 | **1.07** | 0.94 | 1.21 |  | 3,078 | 155 | 5.0 | **1.21** | 1.03 | 1.43 |
|  |  | **>10** | 2,080 | 137 | 6.6 | **1.08** | 0.91 | 1.28 |  | 1,109 | 75 | 6.8 | **1.17** | 0.94 | 1.47 |
|  | **Bedtime** | |  |  |  |  |  |  |  |  |  |  |  |  |  |
|  |  | **21:00–24:00** | 38,579 | 1,974 | 5.1 | **Reference** | |  |  | 35,494 | 1,798 | 5.1 | **Reference** | |  |
|  |  | **24:00–03:00** | 8,123 | 659 | 8.1 | **1.10** | 1.01 | 1.20 |  | 11,816 | 989 | 8.4 | **1.11** | 1.03 | 1.20 |
|  |  | **Other** | 700 | 44 | 6.3 | **1.06** | 0.80 | 1.41 |  | 880 | 57 | 6.5 | **0.99** | 0.77 | 1.29 |
| **Frequent crying, for long periods** | | | | |  |  |  |  |  |  |  |  |  |  |  |
|  | **Sleep duration, hours** | | |  |  |  |  |  |  |  |  |  |  |  |  |
|  |  | **<6** | 693 | 121 | 17.5 | **0.94** | 0.80 | 1.11 |  | 1,588 | 308 | 19.4 | **1.04** | 0.93 | 1.15 |
|  |  | **6–7** | 3,854 | 732 | 19.0 | **1.04** | 0.96 | 1.11 |  | 7,557 | 1,471 | 19.5 | **1.04** | 0.99 | 1.10 |
|  |  | **7–8** | 17,336 | 3,007 | 17.4 | **Reference** | |  |  | 20,788 | 3,607 | 17.4 | **Reference** | |  |
|  |  | **8–9** | 17,419 | 2,678 | 15.4 | **0.97** | 0.92 | 1.02 |  | 13,967 | 2,078 | 14.9 | **0.98** | 0.94 | 1.03 |
|  |  | **9–10** | 5,943 | 868 | 14.6 | **0.96** | 0.90 | 1.03 |  | 3,076 | 400 | 13.0 | **0.90** | 0.82 | 0.99 |
|  |  | **>10** | 2,077 | 354 | 17.0 | **0.99** | 0.90 | 1.10 |  | 1,103 | 195 | 17.7 | **1.03** | 0.91 | 1.18 |
|  | **Bedtime** | |  |  |  |  |  |  |  |  |  |  |  |  |  |
|  |  | **21:00–24:00** | 38,508 | 6,052 | 15.7 | **Reference** | |  |  | 35,420 | 5,517 | 15.6 | **Reference** | |  |
|  |  | **24:00–03:00** | 8,113 | 1,579 | 19.5 | **1.01** | 0.96 | 1.07 |  | 11,778 | 2,395 | 20.3 | **1.05** | 1.00 | 1.10 |
|  |  | **Other** | 701 | 129 | 18.4 | **1.10** | 0.94 | 1.28 |  | 881 | 147 | 16.7 | **0.93** | 0.80 | 1.08 |
| **Intense crying** | | |  |  |  |  |  |  |  |  |  |  |  |  |  |
|  | **Sleep duration, hours** | | |  |  |  |  |  |  |  |  |  |  |  |  |
|  |  | **<6** | 695 | 162 | 23.3 | **1.09** | 0.95 | 1.24 |  | 1,591 | 347 | 21.8 | **1.00** | 0.91 | 1.09 |
|  |  | **6–7** | 3,856 | 813 | 21.1 | **1.01** | 0.94 | 1.08 |  | 7,581 | 1,778 | 23.5 | **1.07** | 1.02 | 1.12 |
|  |  | **7–8** | 17,343 | 3,412 | 19.7 | **Reference** | |  |  | 20,794 | 4,100 | 19.7 | **Reference** | |  |
|  |  | **8–9** | 17,423 | 2,915 | 16.7 | **0.97** | 0.93 | 1.01 |  | 13,972 | 2,227 | 15.9 | **0.98** | 0.93 | 1.02 |
|  |  | **9–10** | 5,941 | 958 | 16.1 | **0.98** | 0.92 | 1.05 |  | 3,078 | 421 | 13.7 | **0.91** | 0.83 | 0.99 |
|  |  | **>10** | 2,070 | 390 | 18.8 | **0.95** | 0.87 | 1.04 |  | 1,103 | 219 | 19.9 | **1.01** | 0.89 | 1.13 |
|  | **Bedtime** | |  |  |  |  |  |  |  |  |  |  |  |  |  |
|  |  | **21:00–24:00** | 38,517 | 6,617 | 17.2 | **Reference** | |  |  | 35,473 | 6,094 | 17.2 | **Reference** | |  |
|  |  | **24:00–03:00** | 8,116 | 1,887 | 23.3 | **1.03** | 0.98 | 1.07 |  | 11,827 | 2,843 | 24.0 | **1.03** | 0.99 | 1.07 |
|  |  | **Other** | 695 | 146 | 21.0 | **1.11** | 0.96 | 1.28 |  | 886 | 178 | 20.1 | **0.96** | 0.84 | 1.09 |
|  |  |  |  |  |  |  |  |  |  |  |  |  |  |  |  |
| CI, confidence interval; RR, relative risk | | | | | | | | | | | | | | | |
| *Adjusted for maternal age at delivery, smoking habits, alcohol consumption, pre-pregnancy body mass index, gestational age at birth, parity, infertility treatment, infant sex, small for gestational age, type of delivery, postpartum depressive symptoms, educational background, household income, and occupation. | | | | | | | | | | | | | | | |
